# Supplementary material for: What do alexithymia items measure? A discriminant content validity study of the Toronto-alexithymia-scale–20
Source: PeerJ. 2021 Jun 29;9:e11639. doi: 10.7717/peerj.11639 (PMC8253111; doi:10.7717/peerj.11639)
Supplement: Supplemental Information 1 [file peerj-09-11639-s001.docx]

**Table S1**

*Toronto Alexithymia Scale - 20 Items Ordered According to the Subscales*

| Items |
| --- |
| *Difficulty identifying feelings scale* |
| 1. I am often confused about what emotion I am feeling. |
| 3. I have physical sensations that even doctors don't understand. |
| 6. When I am upset, I don't know if I am sad, frightened, or angry. |
| 7. I am often puzzled by sensations in my body. |
| 9. I have feelings that I can't quite identify. |
| 13. I don't know what's going on inside me. |
| 14. I often don't know why I am angry. |
| *Difficulty describing feelings scale* |
| 2. It is difficult for me to find the right words for my feelings. |
| 4. I am able to describe my feelings easily. |
| 11. I find it hard to describe how I feel about people. |
| 12. People tell me to describe my feelings more. |
| 17. It is difficult for me to reveal my innermost feelings, even to close friends. |
| *Externally-oriented thinking scale* |
| 5. I prefer to analyze problems rather than just describe them. |
| 8. I prefer to just let things happen rather than to understand why they turned out that way. |
| 10. Being in touch with emotions is essential. |
| 15. I prefer talking to people about their daily activities rather than their feelings. |
| 16. I prefer to watch "light" entertainment shows rather than psychological dramas. |
| 18. I can feel close to someone, even in moments of silence. |
| 19. I find examination of my feelings useful in solving personal problems. |
| 20. Looking for hidden meanings in movies or plays distracts from their enjoyment. |

**Table S2**

*PROMIS® Item Bank v1.0-Emotional Distress-Anxiety – Short Form 4a items*

| Items |
| --- |
| 1. In the past 7 days, I felt fearful. |
| 1. In the past 7 days, I found it hard to focus on anything other than my anxiety. |
| 1. In the past 7 days, my worries overwhelmed me. |
| 1. In the past 7 days, I felt uneasy. |

**Table S3**

*PROMIS® Item Bank v1.0-Emotional Distress-Depression – Short Form 4a items*

| Items |
| --- |
| 1. In the past 7 days, I felt worthless. |
| 1. In the past 7 days, I felt helpless. |
| 1. In the past 7 days, I felt depressed. |
| 1. In the past 7 days, I felt hopeless. |

**Table S4**

*Short Health Anxiety Inventory items*

| Items |
| --- |
| 1. I am always afraid that I have a serious illness. |
| 1. I constantly have images of myself being ill. |
| 1. Nothing can take my mind off thoughts about my health. |
| 1. I usually think that I am seriously ill. |

**Figure S1**

*Estimates and Associated 95% Credibility Intervals of the Relevance Score for each TAS item of the Difficulty Identifying Feelings Scale for each of the Constructs*.


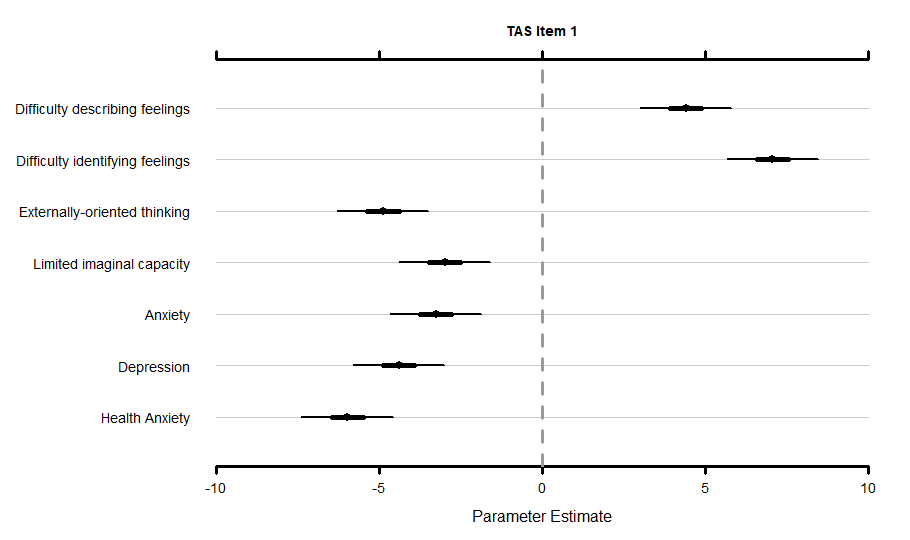

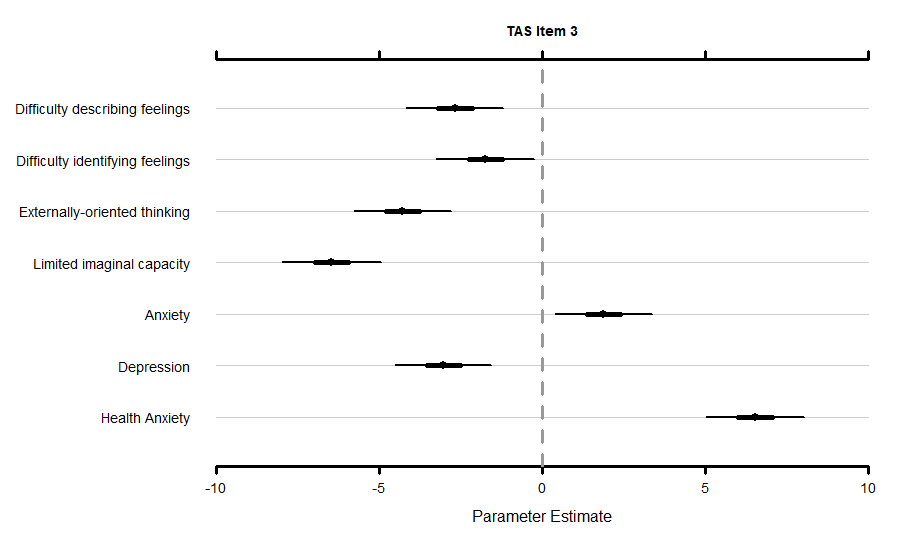

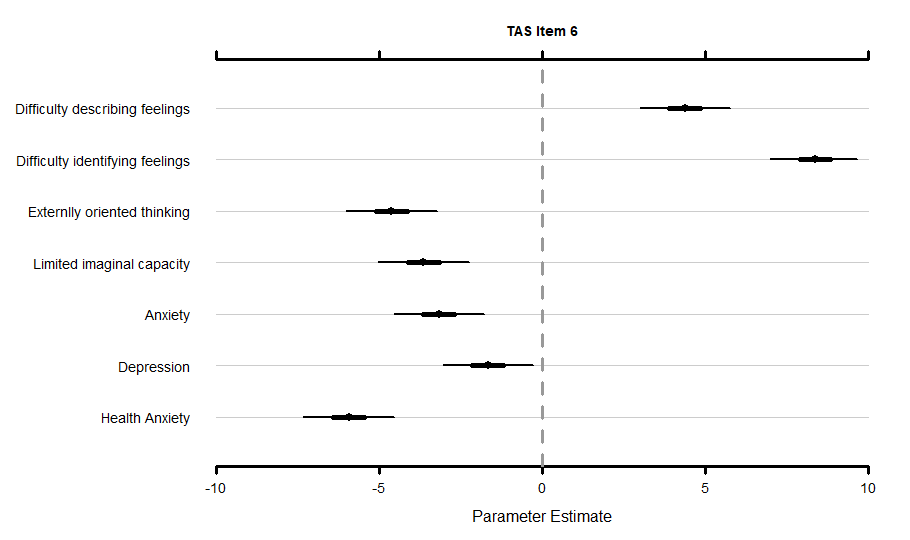

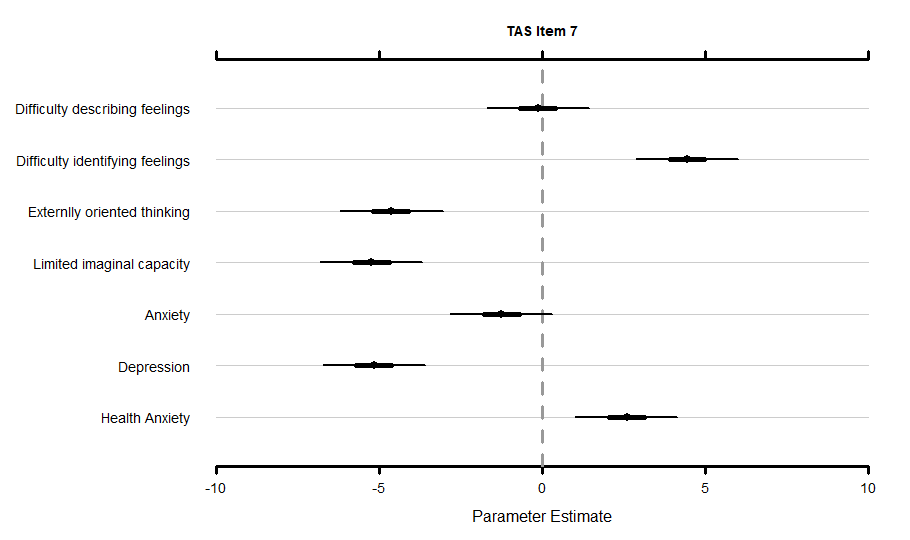

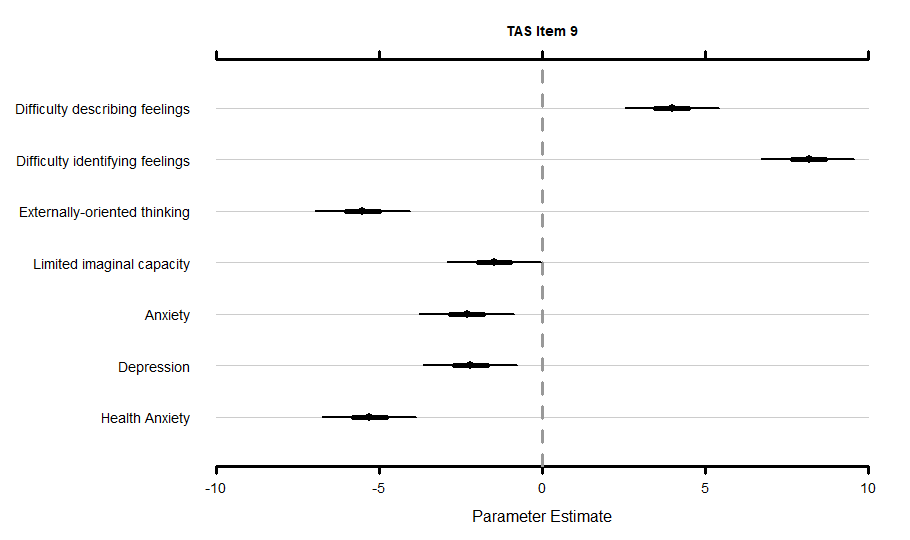

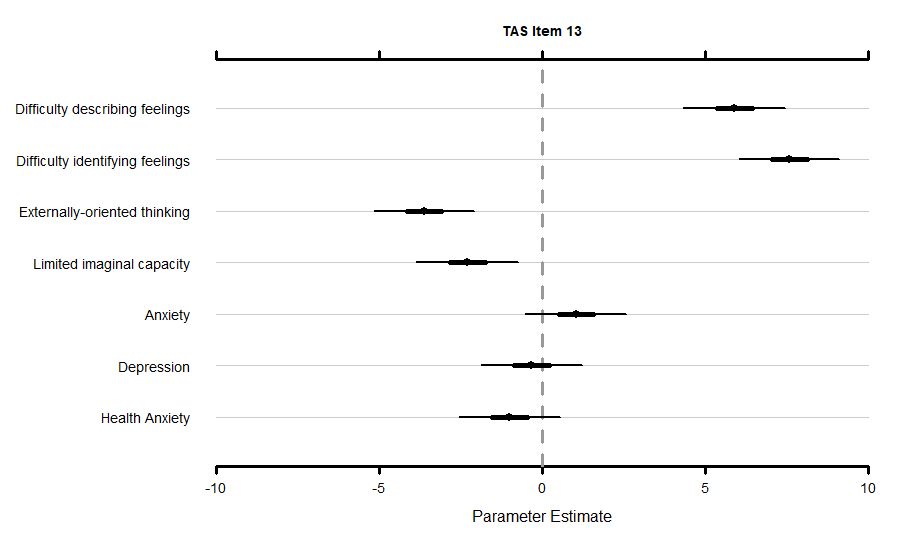

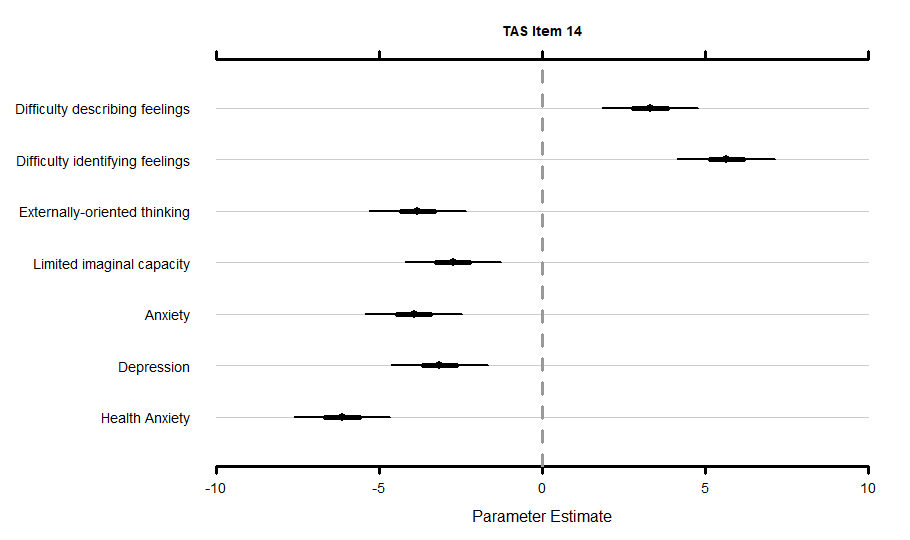


*Note.* TAS = Toronto Alexithymia Scale – 20; PA = PROMIS^®^ Item Bank v1.0-Emotional Distress-Anxiety – Short Form 4a; PD = PROMIS^®^ Item Bank v1.0 – Emotional Distress-Depression – Short Form 4a; SHAI = Short Health Anxiety Inventory.

**Figure S2**

*Estimates and Associated 95% Credibility Intervals of the Relevance Score for each TAS Item of the Difficulty Describing Feelings Scale for each of the Constructs*.


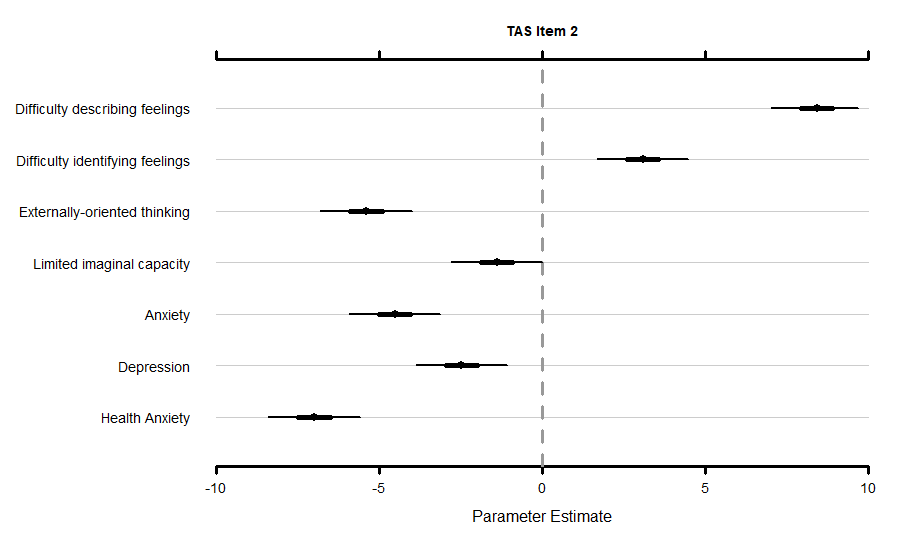

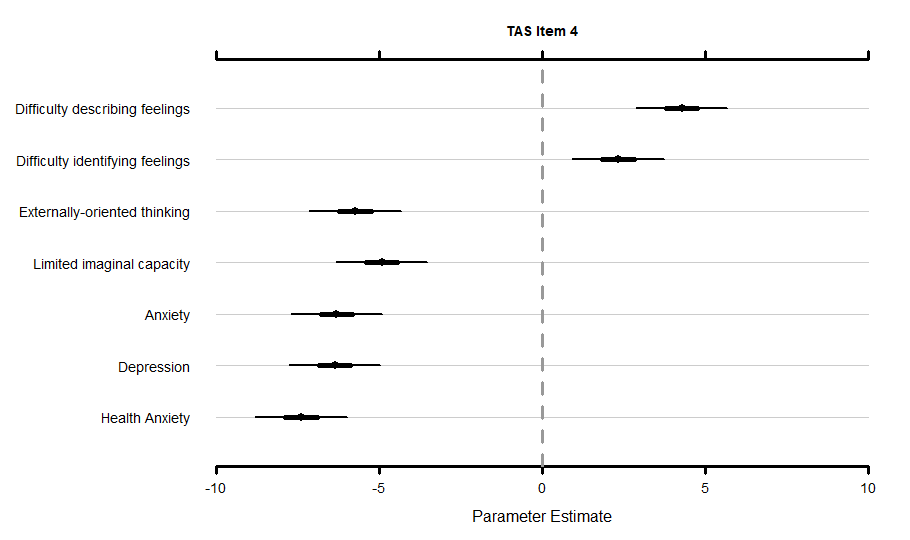

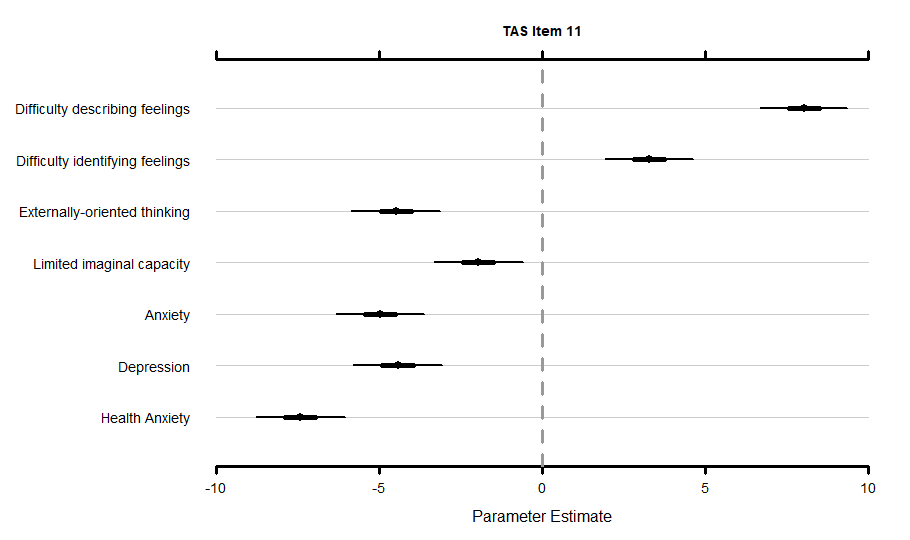

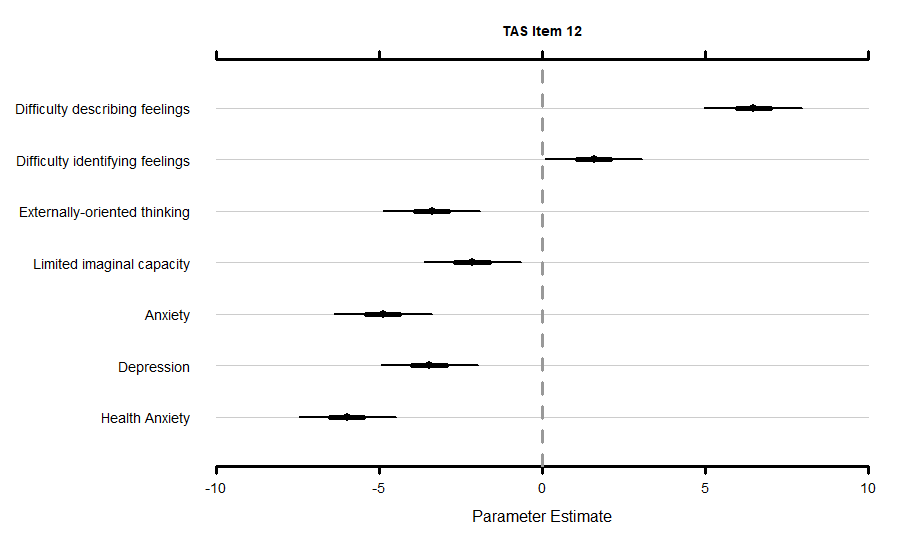

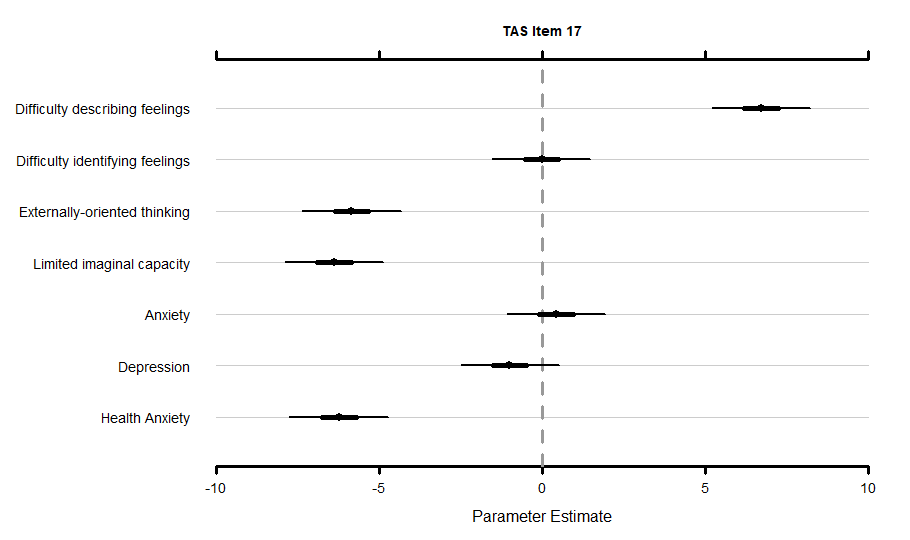


*Note.* TAS = Toronto Alexithymia Scale – 20; PA = PROMIS^®^ Item Bank v1.0-Emotional Distress-Anxiety – Short Form 4a; PD = PROMIS^®^ Item Bank v1.0 – Emotional Distress-Depression – Short Form 4a; SHAI = Short Health Anxiety Inventory.

**Figure S3**

*Estimates and Associated 95% Credibility Intervals of the Relevance Score for each TAS Item of the Externally-Oriented Thinking Scale for each of the Constructs*.


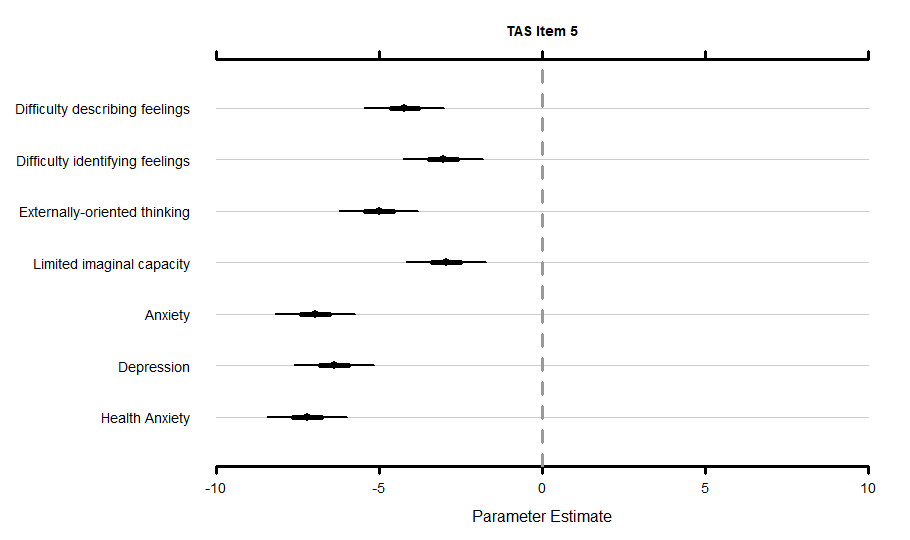

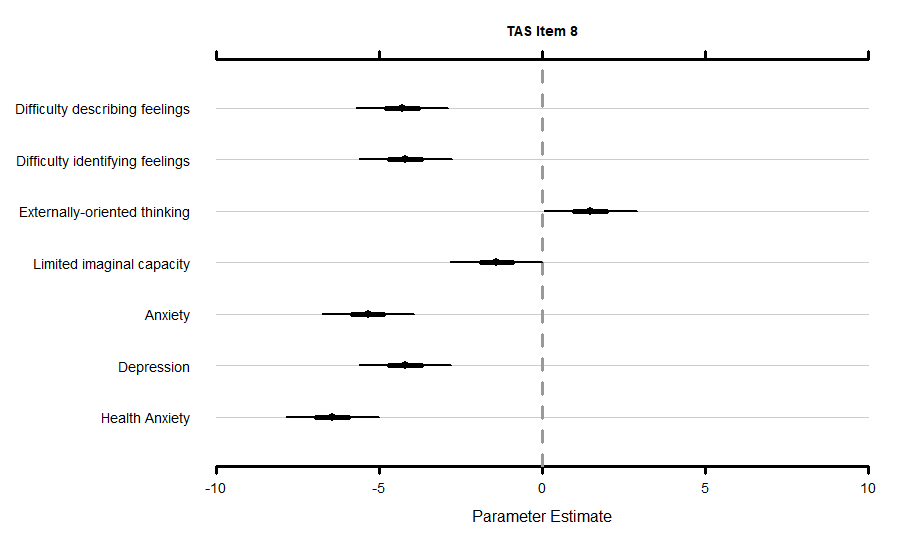

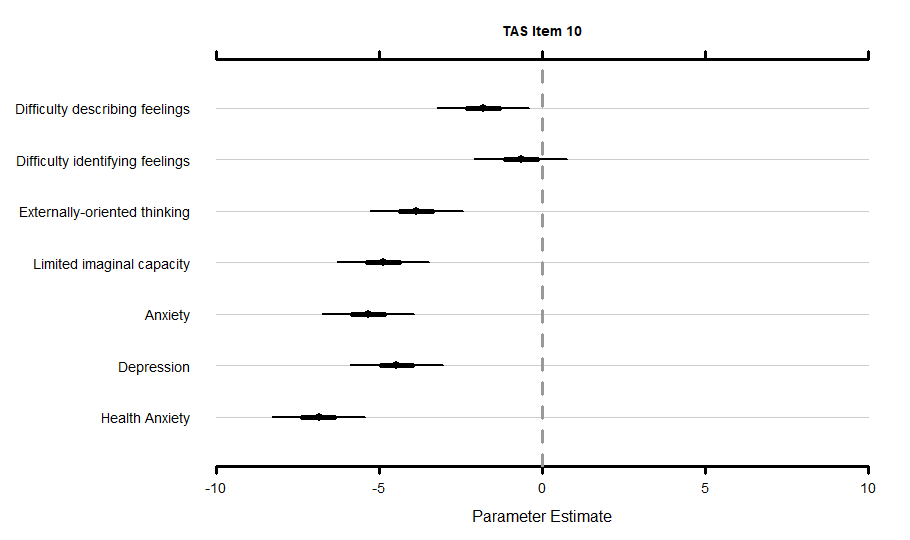

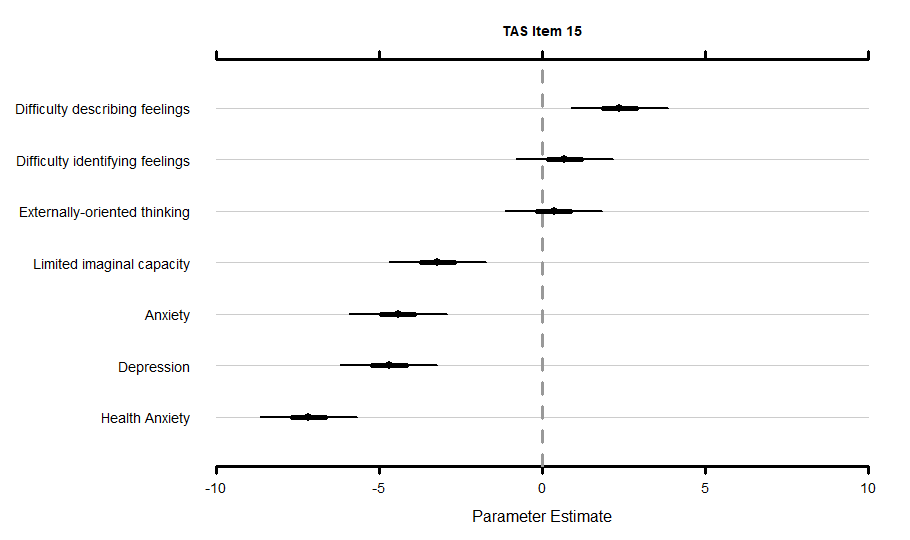

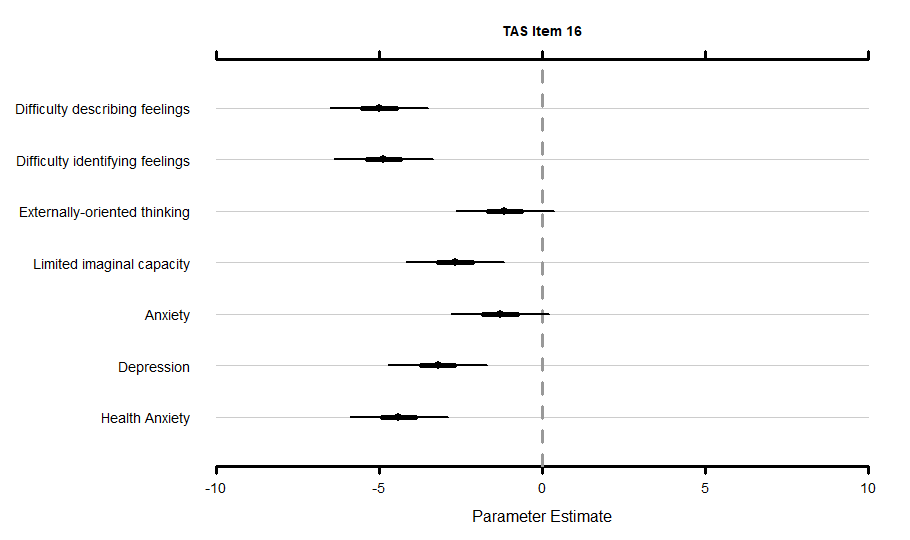

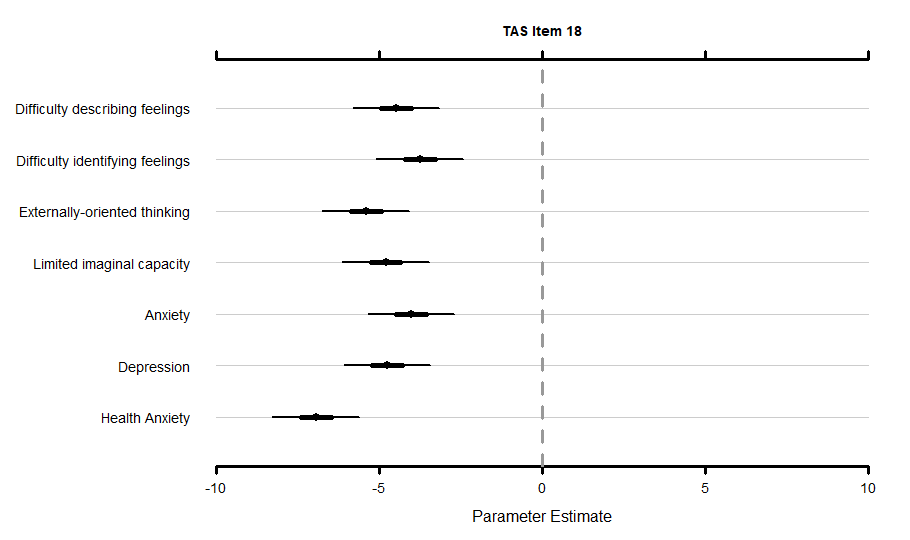

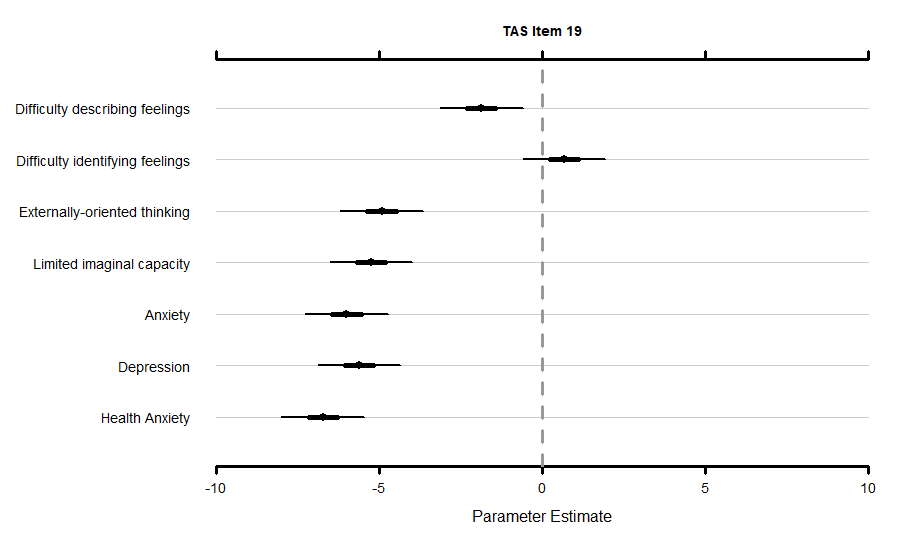

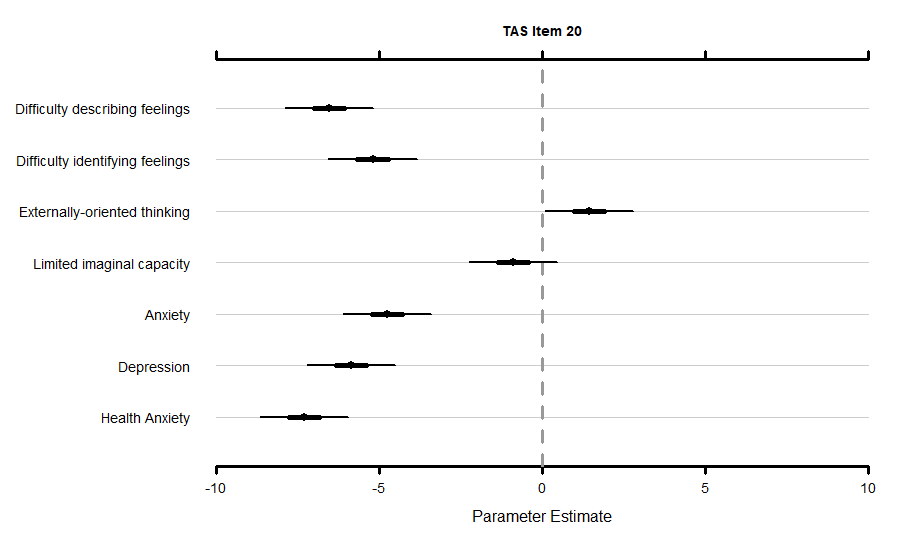


*Note.* TAS = Toronto Alexithymia Scale – 20; PA = PROMIS^®^ Item Bank v1.0-Emotional Distress-Anxiety – Short Form 4a; PD = PROMIS^®^ Item Bank v1.0 – Emotional Distress-Depression – Short Form 4a; SHAI = Short Health Anxiety Inventory.

**Detailed description of the content validity results of the items**

***Item 1***

Item 1, designed to measure difficulty identifying feelings*,* scored highest on ‘difficulty identifying feelings’ ($\hat{\mu}$= 7.08, 95% CI [5.68 to 8.49]). It is not significantly higher than ‘difficulty describing feelings’ ($\hat{\mu}$=4.41, 95% CI [3.01 to 5.82]; Δ = 2.66, 95% CI [0.89 to 4.46]). ‘Difficulty identifying feelings’ is significantly higher than ‘externally-oriented thinking’ ($\hat{\mu}$=-4.89, 95% CI [-6.29 to -3.49]; Δ = 11.97, 95% CI [10.19 to 13.75]), ‘limited imaginal capacity’ ($\hat{\mu}$=-2.99, 95% CI [-4.40 to -1.58]; Δ = 10.06, 95% CI [8.27 to 11.85]), ‘anxiety’ ($\hat{\mu}$=-3.26, 95% CI [-4.66 to -1.87]; Δ = 10.34, 95% CI [8.55 to 12.13]), ‘depression’ ($\hat{\mu}$=-4.41, 95% CI [-5.81 to -3.00]; Δ = 11.49, 95% CI [9.72 to 13.28]), and ‘health anxiety’ ($\hat{\mu}$=-5.99, 95% CI [-7.39 to -4.59]; Δ = 13.06, 95% CI [11.29 to 14.84]).

***Item 2***

Item 2, designed to measure difficulty describing feelings, scored significantly higher on ‘difficulty describing feelings’ ($\hat{\mu}$= 8.42, 95% CI [7.04 to 9.70]), compared to ‘difficulty identifying feelings’ ($\hat{\mu}$=3.09, 95% CI [1.69 to 4.50]; Δ = 5.32, 95% CI [3.52 to 7.07]), ‘externally-oriented thinking’ ($\hat{\mu}$=-5.41, 95% CI [-6.82 to -4.00]; Δ = 13.83, 95% CI [12.03 to 15.59]), ‘limited imaginal capacity’ ($\hat{\mu}$=-1.39, 95% CI [-2.80 to 0.01]; Δ = 9.81, 95% CI [8.01 to 11.57]), ‘anxiety’ ($\hat{\mu}$=-4.52, 95% CI [-5.93 to -3.13]; Δ = 12.94, 95% CI [11.15 to 14.69]), ‘depression’ ($\hat{\mu}$=-2.48, 95% CI [-3.88 to -1.07]; Δ = 10.90, 95% CI [9.08 to 12.66]), and ‘health anxiety’ ($\hat{\mu}$=-7.00, 95% CI [-8.41 to -5.59]; Δ = 15.42, 95% CI [13.63 to 17.19]).

***Item 3***

Item 3, designed to measure difficulty identifying feelings, scored significantly higher on ‘health anxiety’ ($\hat{\mu}$= 6.54, 95% CI [5.02 to 8.04]), compared to ‘difficulty describing feelings’ ($\hat{\mu}$=-2.68, 95% CI [-4.19 to -1.19]; Δ = 9.21, 95% CI [7.18 to 11.24]), ‘difficulty identifying feelings’ ($\hat{\mu}$=-1.74, 95% CI [-3.24 to -0.25]; Δ = 8.28, 95% CI [6.24 to 10.29]), ‘externally-oriented thinking’ ($\hat{\mu}$=-4.29, 95% CI [-5.79 to -2.80]; Δ = 10.83, 95% CI [8.80 to 12.84]), ‘limited imaginal capacity’ ($\hat{\mu}$=-6.47, 95% CI [-7.98 to -4.96]; Δ = 13.01, 95% CI [10.98 to 15.04]), ‘anxiety’ ($\hat{\mu}$=1.88, 95% CI [0.39 to 3.38]; Δ = 4.66, 95% CI [2.65 to 6.68]), and ‘depression’ ($\hat{\mu}$=-3.03, 95% CI [-4.53 to -1.55]; Δ = 9.57, 95% CI [7.54 to 11.59]). The score for ‘difficulty identifying feelings’ ($\hat{\mu}$=-1.74, 95% CI [-3.24 to -0.25]) is not significantly different from ‘difficulty describing feelings’ ($\hat{\mu}$=-2.68, 95% CI [-4.19 to -1.19]; Δ = 0.94, 95% CI [-1.08 to 2.97]), or ‘externally-oriented thinking’ ($\hat{\mu}$=-4.29, 95% CI [-5.79 to -2.80]; Δ = 2.55, 95% CI [0.53 to 4.55]). It is significantly higher compared to ‘limited imaginal capacity’ ($\hat{\mu}$=-6.47, 95% CI [-7.98 to -4.96]; Δ = 4.73, 95% CI [2.71 to 6.76]), significantly lower compared to ‘anxiety’ ($\hat{\mu}$=1.88, 95% CI [0.39 to 3.38]; Δ = -3.62, 95% CI [-5.65 to -1.61]), not significantly different from ‘depression’ ($\hat{\mu}$=-3.03, 95% CI [-4.53 to -1.55]; Δ = 1.29, 95% CI [-0.72 to 3.31]), and significantly lower compared to ‘health anxiety’ ($\hat{\mu}$= 6.54, 95% CI [5.02 to 8.04]; Δ = -8.28, 95% CI [-10.29 to -6.24]).

***Item 4***

Item 4, designed to measure difficulty describing feelings, scored highest on ‘difficulty describing feelings’ ($\hat{\mu}$= 4.29, 95% CI [2.89 to 5.69]). It is not significantly higher than ‘difficulty identifying feelings’ ($\hat{\mu}$=2.32, 95% CI [0.92 to 3.75]; Δ = 1.97, 95% CI [0.14 to 3.80]). ‘Difficulty describing feelings’ is significantly higher compared to ‘externally-oriented thinking’ ($\hat{\mu}$=-5.74, 95% CI [-7.15 to -4.33]; Δ = 10.03, 95% CI [8.21 to 11.85]), ‘limited imaginal capacity’ ($\hat{\mu}$=-4.92, 95% CI [-6.33 to -3.52]; Δ = 9.21, 95% CI [7.39 to 11.05]), ‘anxiety’ ($\hat{\mu}$=-6.32, 95% CI [-7.72 to -4.92]; Δ = 10.61, 95% CI [8.81 to 12.43]), ‘depression’ ($\hat{\mu}$=-6.37, 95% CI [-7.78 to -4.98]; Δ = 10.66, 95% CI [8.84 to 12.49]), and ‘health anxiety’ ($\hat{\mu}$=-7.40, 95% CI [-8.81 to -5.99]; Δ = 11.69, 95% CI [9.87 to 13.51]).

***Item 5***

Item 5, designed to measure externally-oriented thinking, scored highest on ‘limited imaginal capacity’. Its score on ‘externally-oriented thinking’ ($\hat{\mu}$= -5.01, 95% CI [-6.22 to -3.80]), is not significantly different from ‘difficulty describing feelings’ ($\hat{\mu}$=-4.23, 95% CI [-5.46 to -3.00]; Δ = -0.78, 95% CI [-2.31 to 0.75]), or ‘difficulty identifying feelings’ ($\hat{\mu}$=-3.04, 95% CI [-4.27 to -1.81]; Δ = -1.97, 95% CI [-3.50 to -0.43]), or ‘limited imaginal capacity’ ($\hat{\mu}$=-2.95, 95% CI [-4.17 to -1.73]; Δ = -2.06, 95% CI [-3.59 to -0.53]). It is significantly higher compared to ‘anxiety’ ($\hat{\mu}$=-6.97, 95% CI [-8.21 to -5.75]; Δ = 1.96, 95% CI [0.42 to 3.48]), ‘depression’ ($\hat{\mu}$=-6.39, 95% CI [-7.62 to -5.16]; Δ = 1.38, 95% CI [-0.16 to 2.90]), and ‘health anxiety’ ($\hat{\mu}$=-7.22, 95% CI [-8.45 to -5.99]; Δ = -1.97, 95% CI [-3.50 to -0.43]).

***Item 6***

Item 6, designed to measure difficulty identifying feelings, scored significantly higher on ‘difficulty identifying feelings’ ($\hat{\mu}$= 8.36, 95% CI [6.99 to 9.66]), compared to ‘difficulty describing feelings’ ($\hat{\mu}$=4.38, 95% CI [2.99 to 5.77]; Δ = 3.99, 95% CI [2.27 to 5.67]), ‘externally-oriented thinking’ ($\hat{\mu}$=-4.64, 95% CI [-6.04 to -3.25]; Δ = 13.01, 95% CI [11.29 to 14.69]), ‘limited imaginal capacity’ ($\hat{\mu}$=-3.64, 95% CI [-5.03 to -2.25]; Δ = 12.00, 95% CI [10.28 to 13.70]), ‘anxiety’ ($\hat{\mu}$=-3.19, 95% CI [-4.57 to -1.79]; Δ = 11.55, 95% CI [9.83 to 13.25]), ‘depression’ ($\hat{\mu}$=-1.68, 95% CI [-3.06 to -0.29]; Δ = 10.04, 95% CI [8.30 to 11.74]), and ‘health anxiety’ ($\hat{\mu}$=-5.94, 95% CI [-7.33 to -4.55]; Δ = 14.31, 95% CI [12.57 to 16.00]).

***Item 7***

Item 7, designed to measure difficulty identifying feelings, scored significantly higher on ‘difficulty identifying feelings’ ($\hat{\mu}$= 4.45, 95% CI [2.88 to 6.01]), compared to ‘difficulty describing feelings’ ($\hat{\mu}$=-0.13, 95% CI [-1.68 to 1.43]; Δ = 4.58, 95% CI [2.52 to 6.64]), ‘externally-oriented thinking’ ($\hat{\mu}$=-4.63, 95% CI [-6.19 to -3.07]; Δ = 9.08, 95% CI [7.01 to 11.16]), ‘limited imaginal capacity’ ($\hat{\mu}$=-5.24, 95% CI [-6.80 to -3.67]; Δ = 9.69, 95% CI [7.63 to 11.75]), ‘anxiety’ ($\hat{\mu}$=-1.25, 95% CI [-2.81 to 0.32]; Δ = 5.70, 95% CI [3.62 to 7.75]), ‘depression’ ($\hat{\mu}$=-5.17, 95% CI [-6.74 to -3.61]; Δ = 9.62, 95% CI [7.55 to 11.69]), and not significantly compared to ‘health anxiety’ ($\hat{\mu}$=2.59 , 95% CI [1.03 to 4.15]; Δ = 1.85, 95% CI [-0.20 to 3.92]).

***Item 8***

Item 8, designed to measure externally-oriented thinking, scored significantly higher on ‘externally-oriented thinking’ ($\hat{\mu}$= 1.48, 95% CI [0.06 to 2.91]), compared to ‘difficulty describing feelings’ ($\hat{\mu}$=-4.30, 95% CI [-5.72 to -2.88]; Δ = 5.78, 95% CI [4.02 to 7.55]), ‘difficulty identifying feelings’ ($\hat{\mu}$=-4.20, 95% CI [-5.62 to -2.77]; Δ = 5.68, 95% CI [3.90 to 7.45]), ‘limited imaginal capacity’ ($\hat{\mu}$=-1.40, 95% CI [-2.82 to 0.01]; Δ = 2.88, 95% CI [1.13 to 4.65]), ‘anxiety’ ($\hat{\mu}$=-5.36, 95% CI [-6.76 to -3.94]; Δ = 6.84, 95% CI [5.08 to 8.60]), ‘depression’ ($\hat{\mu}$=-4.20, 95% CI [-5.62 to -2.78]; Δ = 5.68, 95% CI [3.90 to 7.46]), and ‘health anxiety’ ($\hat{\mu}$=-6.45, 95% CI [-7.86 to -5.01]; Δ = 7.93, 95% CI [6.17 to 9.69]).

***Item 9***

Item 9, designed to measure difficulty identifying feelings, scored significantly higher on ‘difficulty identifying feelings’ ($\hat{\mu}$= 8.17, 95% CI [6.73 to 9.57]), compared to ‘difficulty describing feelings’ ($\hat{\mu}$=3.99, 95% CI [2.54 to 5.45]; Δ = 4.19, 95% CI [2.38 to 5.97]), ‘externally-oriented thinking’ ($\hat{\mu}$=-5.52, 95% CI [-6.97 to -4.07]; Δ = 13.69, 95% CI [11.87 to 15.47]), ‘limited imaginal capacity’ ($\hat{\mu}$=-1.47, 95% CI [-2.91 to -0.02]; Δ = 9.64, 95% CI [7.83 to 11.41]), ‘anxiety’ ($\hat{\mu}$=-2.31, 95% CI [-3.77 to -0.86]; Δ = 10.49, 95% CI [8.69 to 12.27]), ‘depression’ ($\hat{\mu}$=-2.20, 95% CI [-3.66 to -0.77]; Δ = 10.37, 95% CI [8.56 to 12.18]), and ‘health anxiety’ ($\hat{\mu}$=-5.30, 95% CI [-6.75 to -3.87]) (Δ = 13.47, 95% CI [11.66 to 15.26]).

***Item 10***

Item 10, designed to measure externally-oriented thinking, scored significantly higher on ‘difficulty identifying feelings’ ($\hat{\mu}$= -0.65, 95% CI [-2.08 to 0.77]), compared to ‘difficulty describing feelings’ ($\hat{\mu}$=-1.81, 95% CI [-3.23 to -0.39]; Δ = 1.16, 95% CI [-0.57 to 2.92]), ‘externally-oriented thinking’ ($\hat{\mu}$=-3.86, 95% CI [-5.28 to -2.44]; Δ = 3.21, 95% CI [1.46 to 4.95]), ‘limited imaginal capacity’ ($\hat{\mu}$=-4.88, 95% CI [-6.31 to -3.46]; Δ = 4.23, 95% CI [2.49 to 5.97]), ‘anxiety’ ($\hat{\mu}$=-5.34, 95% CI [-6.75 to -3.92]; Δ = 4.69, 95% CI [2.94 to 6.44]), ‘depression’ ($\hat{\mu}$=-4.47, 95% CI [-5.89 to -3.05]) (Δ = 3.82, 95% CI [2.08 to 5.58]), and ‘health anxiety’ ($\hat{\mu}$=-6.87, 95% CI [-8.30 to -5.44]) (Δ = 6.22, 95% CI [4.47 to 7.96]). Its score on ‘externally-oriented thinking’ ($\hat{\mu}$= -3.86, 95% CI [-5.28 to -2.44]), is significantly lower compared to ‘difficulty describing feelings’ ($\hat{\mu}$=-1.81, 95% CI [-3.23 to -0.39]) (Δ = -2.05, 95% CI [-3.80 to -0.30]), significantly lower compared to ‘difficulty identifying feelings’ ($\hat{\mu}$=-0.65, 95% CI [-2.08 to 0.77]; Δ = 0.61, 95% CI [-1.13 to 2.34]), not significantly different from ‘limited imaginal capacity’ ($\hat{\mu}$=-4.88, 95% CI [-6.31 to -3.46]; Δ = 1.01, 95% CI [-0.74 to 2.76]), not significantly different from ‘anxiety’ ($\hat{\mu}$=-5.34, 95% CI [-6.75 to -3.92]; Δ = 1.47, 95% CI [-0.27 to 3.20]), not significantly different from ‘depression’ ($\hat{\mu}$=-4.47, 95% CI [-5.89 to -3.05]; Δ = 0.61, 95% CI [-1.13 to 2.34]), and significantly higher compared to ‘health anxiety’ ($\hat{\mu}$=-6.87, 95% CI [-8.30 to -5.44]; Δ = 0.61, 95% CI [-1.13 to 2.34]).

***Item 11***

Item 11, designed to measure difficulty describing feelings, scored significantly higher on ‘difficulty describing feelings’ ($\hat{\mu}$= 8.04, 95% CI [6.68 to 9.37]), compared to ‘difficulty identifying feelings’ ($\hat{\mu}$=3.29, 95% CI [1.94 to 4.64]; (Δ = 4.75, 95% CI [2.96 to 6.54]), ‘externally-oriented thinking’ ($\hat{\mu}$=-4.49, 95% CI [-5.85 to -3.13]; Δ = 12.53, 95% CI [10.74 to 14.30]), ‘limited imaginal capacity’ ($\hat{\mu}$=-1.97, 95% CI [-3.33 to -0.60]; Δ = 10.01, 95% CI [8.22 to 11.79]), ‘anxiety’ ($\hat{\mu}$=-4.98, 95% CI [-6.34 to -3.62]; Δ = 13.02, 95% CI [11.24 to 14.79]), ‘depression’ ($\hat{\mu}$=-4.43, 95% CI [-5.81 to -3.06]; Δ = 12.47, 95% CI [10.69 to 14.25]), and ‘health anxiety’ ($\hat{\mu}$=-7.43, 95% CI [-8.79 to -6.06]; Δ = 15.47, 95% CI [13.68 to 17.24]).

***Item 12***

Item 12, designed to measure difficulty describing feelings, scored significantly higher on ‘difficulty describing feelings’ ($\hat{\mu}$= 6.49, 95% CI [4.99 to 7.99]), compared to ‘difficulty identifying feelings’ ($\hat{\mu}$=1.58, 95% CI [0.09 to 3.08]; Δ = 4.90, 95% CI [3.01 to 6.78]), ‘externally-oriented thinking’ ($\hat{\mu}$=-3.38, 95% CI [-4.88 to -1.90]; Δ = 9.87, 95% CI [7.98 to 11.76]), ‘limited imaginal capacity’ ($\hat{\mu}$=-2.15, 95% CI [-3.64 to -0.65]; Δ = 8.63, 95% CI [6.74 to 10.53]), ‘anxiety’ ($\hat{\mu}$=-4.90, 95% CI [-6.39 to -3.38]; Δ = 11.38, 95% CI [9.50 to 13.27]), ‘depression’ ($\hat{\mu}$=-3.46, 95% CI [-4.95 to -1.97]; Δ = 9.95, 95% CI [8.05 to 11.85]), and ‘health anxiety’ ($\hat{\mu}$=-5.99, 95% CI [-7.47 to -4.50]; Δ = 12.48, 95% CI [10.58 to 14.36]).

***Item 13***

Item 13, designed to measure difficulty identifying feelings, scored highest on ‘difficulty identifying feelings’ ($\hat{\mu}$= 7.60, 95% CI [6.04 to 9.13]). It is not significantly higher compared to ‘difficulty describing feelings’ ($\hat{\mu}$=5.90, 95% CI [4.33 to 7.46]; Δ = 1.69, 95% CI [-0.28 to 3.66]). ‘Difficulty identifying feelings’ scores significantly higher compared to ‘externally-oriented thinking’ ($\hat{\mu}$=-3.62, 95% CI [-5.17 to -2.07]; Δ = 11.22, 95% CI [9.26 to 13.18]), ‘limited imaginal capacity’ ($\hat{\mu}$=-2.30, 95% CI [-3.86 to -0.74]; Δ = 9.90, 95% CI [7.93 to 11.85]), ‘anxiety’ ($\hat{\mu}$=1.04, 95% CI [-0.51 to 2.59]; Δ = 6.56, 95% CI [4.59 to 8.51]), ‘depression’ ($\hat{\mu}$=-0.33, 95% CI [-1.88 to 1.22]; Δ = 7.92, 95% CI [5.96 to 9.89]), and ‘health anxiety’ ($\hat{\mu}$=-1.01, 95% CI [-2.56 to 0.55]; Δ = 8.60, 95% CI [6.64 to 10.55]).

***Item 14***

Item 14, designed to measure difficulty identifying feelings, scored highest on ‘difficulty identifying feelings’ ($\hat{\mu}$= 5.66, 95% CI [4.16 to 7.16]). It is not significantly higher than ‘difficulty describing feelings’ ($\hat{\mu}$=3.32, 95% CI [1.83 to 4.81]; Δ = 2.35, 95% CI [0.56 to 4.15]). ‘Difficulty identifying feelings’ scores significantly higher compared to ‘externally-oriented thinking’ ($\hat{\mu}$=-3.83, 95% CI [-5.31 to -2.34]; Δ = 9.49, 95% CI [7.68 to 11.29]), ‘limited imaginal capacity’ ($\hat{\mu}$=-2.74, 95% CI [-4.22 to -1.26]; Δ = 8.40, 95% CI [6.61 to 10.21]), ‘anxiety’ ($\hat{\mu}$=-3.94, 95% CI [-5.43 to -2.45]; Δ = 9.61, 95% CI [7.82 to 11.42]), ‘depression’ ($\hat{\mu}$=-3.16, 95% CI [-4.64 to -1.67]; Δ = 8.82, 95% CI [7.02 to 10.62]), and ‘health anxiety’ ($\hat{\mu}$=-6.14, 95% CI [-7.62 to -4.66]; Δ = 11.81, 95% CI [10.02 to 13.62]).

***Item 15***

Item 15, designed to measure externally-oriented thinking, scored highest on ‘difficulty describing feelings*’,* ($\hat{\mu}$= 2.38, 95% CI [0.88 to 3.86]). It is not significantly compared to ‘difficulty identifying feelings’ ($\hat{\mu}$=0.69, 95% CI [-0.81 to 2.19]; Δ = 1.68, 95% CI [-0.18 to 3.54]) and ‘externally-oriented thinking’ ($\hat{\mu}$=0.36, 95% CI [-1.15 to 1.86]; Δ = 2.02, 95% CI [0.15 to 3.87]). ‘Difficulty describing feelings’ scored significantly higher compared to ‘limited imaginal capacity’ ($\hat{\mu}$=-3.21, 95% CI [-4.71 to -1.72]; Δ = 5.59, 95% CI [3.72 to 7.47]), ‘anxiety’ ($\hat{\mu}$=-4.43 , 95% CI [-5.92 to -2.93]; Δ = 6.81, 95% CI [4.96 to 8.66]), ‘depression’ ($\hat{\mu}$=-4.70, 95% CI [-6.19 to -3.21]; Δ = 7.08, 95% CI [5.20 to 8.94]), and ‘health anxiety’ ($\hat{\mu}$=-7.17, 95% CI [-8.66 to -5.68]; Δ = 9.55, 95% CI [7.69 to 11.41]). Its score on ‘externally-oriented thinking’ ($\hat{\mu}$= 0.36, 95% CI [-1.15 to 1.86]), is not significantly different from ‘difficulty describing feelings’ ($\hat{\mu}$=2.38, 95% CI [0.88 to 3.87]; Δ = -2.02, 95% CI [-3.87 to -0.15]), or ‘difficulty identifying feelings’ ($\hat{\mu}$=0.69, 95% CI [-0.81 to 2.19]; Δ = -0.34, 95% CI [-2.21 to 1.54]). It significantly higher compared to ‘limited imaginal capacity’ ($\hat{\mu}$=-3.21, 95% CI [-4.71 to -1.72]; Δ = 3.57, 95% CI [1.70 to 5.44]), ‘anxiety’ ($\hat{\mu}$=-4.43, 95% CI [-5.92 to -2.93]; Δ = 4.79, 95% CI [2.91 to 6.65]), ‘depression’ ($\hat{\mu}$=-4.70, 95% CI [-6.19 to -3.21]; Δ = 5.06, 95% CI [3.19 to 6.93]), and ‘health anxiety’ ($\hat{\mu}$=-7.17, 95% CI [-8.66 to -5.68]; Δ = 7.53, 95% CI [5.67 to 9.39]).

***Item 16***

Item 16, designed to measure externally-oriented thinking, scored significantly higher on ‘externally-oriented thinking’ ($\hat{\mu}$= -1.15, 95% CI [-2.66 to 0.36]), compared to ‘difficulty describing feelings’ ($\hat{\mu}$=-5.02, 95% CI [-6.52 to -3.50]; Δ = 3.86, 95% CI [2.11 to 5.60]), ‘difficulty identifying feelings’ ($\hat{\mu}$=-4.87, 95% CI [-6.38 to -3.36]; Δ = -2.20, 95% CI [-3.94 to -0.46]), not significantly different from ‘limited imaginal capacity’ ($\hat{\mu}$=-2.67, 95% CI [-4.17 to -1.15]; Δ = 1.52, 95% CI [-0.22 to 3.25]), not significantly different from ‘anxiety’ ($\hat{\mu}$=-1.30, 95% CI [-2.81 to 0.21]; Δ = 0.14, 95% CI [-1.61 to 1.88]), ‘depression’ ($\hat{\mu}$=-3.21, 95% CI [-4.73 to -1.70]; Δ = -2.20, 95% CI [-3.94 to -0.46]), and significantly higher compared to ‘health anxiety’ ($\hat{\mu}$=-4.41, 95% CI [-5.91 to -2.89]; Δ = -2.20, 95% CI [-3.94 to -0.46]).

***Item 17***

Item 17, designed to measure difficulty describing feelings, scored significantly higher on ‘difficulty describing feelings’ ($\hat{\mu}$= 6.72, 95% CI [5.22 to 8.24]), compared to ‘difficulty identifying feelings’ ($\hat{\mu}$=-0.01, 95% CI [-1.53 to 1.48]; Δ = 6.74, 95% CI [4.86 to 8.62]), ‘externally-oriented thinking’ ($\hat{\mu}$=-5.86, 95% CI [-7.36 to -4.34]; Δ = 12.58, 95% CI [10.70 to 14.48]), ‘limited imaginal capacity’ ($\hat{\mu}$=-6.39, 95% CI [-7.89 to -4.88]; Δ = 13.11, 95% CI [11.22 to 15.00]), ‘anxiety’ ($\hat{\mu}$=0.42, 95% CI [-1.08 to 1.93]; Δ = 6.30, 95% CI [4.43 to 8.19]), ‘depression’ ($\hat{\mu}$=-1.00, 95% CI [-2.49 to 0.51]; Δ = 7.73, 95% CI [5.85 to 9.61]), and ‘health anxiety’ ($\hat{\mu}$=-6.24, 95% CI [-7.76 to -4.74]; Δ = 12.97, 95% CI [11.08 to 14.85]).

***Item 18***

Item 18, designed to measure externally-oriented thinking, scored highest on ‘difficulty identifying feelings’ ($\hat{\mu}$= -3.75, 95% CI [-5.09 to -2.43]). Its score on ‘externally-oriented thinking’ ($\hat{\mu}$= -5.41, 95% CI [-6.75 to -4.08]), is not significantly different from ‘difficulty describing feelings’ ($\hat{\mu}$=-4.49, 95% CI [-5.81 to -3.15]; Δ = -0.93, 95% CI [-2.60 to 0.75]), ‘difficulty identifying feelings’ ($\hat{\mu}$=-3.75 , 95% CI [-5.09 to -2.43]; Δ = 3.20, 95% CI [1.51 to 4.87]), ‘limited imaginal capacity’ ($\hat{\mu}$=-4.80 , 95% CI [-6.14 to -3.47]; Δ = -0.61, 95% CI [-2.28 to 1.09]), ‘anxiety’ ($\hat{\mu}$=-4.02, 95% CI [-5.36 to -2.70]; Δ = -1.39, 95% CI [-3.06 to 0.28]), ‘depression’ ($\hat{\mu}$=-4.76, 95% CI [-6.08 to -3.43]; Δ = 3.20, 95% CI [1.51 to 4.87]), and ‘health anxiety’ ($\hat{\mu}$=-6.95, 95% CI [-8.28 to -5.62]; Δ = 3.20, 95% CI [1.51 to 4.87]).

***Item 19***

Item 19, designed to measure externally-oriented thinking, scored significantly higher on ‘difficulty identifying feelings’ ($\hat{\mu}$= 0.67, 95% CI [-0.59 to 1.94]), compared to ‘difficulty describing feelings’ ($\hat{\mu}$=-1.86, 95% CI [-3.13 to -0.59]; Δ = 2.53, 95% CI [0.93 to 4.13]), ‘externally-oriented thinking’ ($\hat{\mu}$=-4.92, 95% CI [-6.19 to -3.64]; Δ = 5.59, 95% CI [3.99 to 7.20]), ‘limited imaginal capacity’ ($\hat{\mu}$=-5.26, 95% CI [-6.52 to -3.98]; Δ = 5.93, 95% CI [4.33 to 7.54]), ‘anxiety’ ($\hat{\mu}$=-6.00, 95% CI [-7.27 to -4.74]; Δ = 6.68, 95% CI [5.08 to 8.27]), ‘depression’ ($\hat{\mu}$=-5.61, 95% CI [-6.88 to -4.36]; Δ = 6.29, 95% CI [4.69 to 7.89]), and ‘health anxiety’ ($\hat{\mu}$=-6.73, 95% CI [-8.00 to -5.48]; Δ = 7.40, 95% CI [5.81 to 9.01]). Its score on ‘externally-oriented thinking’ ($\hat{\mu}$= -4.92, 95% CI [-6.19 to -3.64]), is significantly lower compared to ‘difficulty describing feelings’ ($\hat{\mu}$=-1.86, 95% CI [-3.13 to -0.59]; Δ = -3.06, 95% CI [-4.65 to -1.46]), significantly lower compared to ‘difficulty identifying feelings’ ($\hat{\mu}$=0.67, 95% CI [-0.59 to 1.94]; Δ = 2.53, 95% CI [0.93 to 4.13]), not significantly different from ‘limited imaginal capacity’ ($\hat{\mu}$=-5.26, 95% CI [-6.52 to -3.98]; Δ = 0.34, 95% CI [-1.27 to 1.94]), not significantly different from ‘anxiety’ ($\hat{\mu}$=-6.00, 95% CI [-7.27 to -4.74]; Δ = 1.08, 95% CI [-0.52 to 2.67]), not significantly different from ‘depression’ ($\hat{\mu}$=-5.61, 95% CI [-6.88 to -4.36]; Δ = 2.53, 95% CI [0.93 to 4.13]), and not significantly different from ‘health anxiety’ ($\hat{\mu}$=-6.73, 95% CI [-8.00 to -5.48]; Δ = 2.53, 95% CI [0.93 to 4.13]).

***Item 20***

Item 20, designed to measure externally-oriented thinking, scored significantly higher on ‘externally-oriented thinking’ ($\hat{\mu}$= 1.45, 95% CI [0.09 to 2.79]), compared to ‘difficulty describing feelings’ ($\hat{\mu}$=-6.53, 95% CI [-7.89 to -5.19]; Δ = 7.98, 95% CI [6.15 to 9.82]), ‘difficulty identifying feelings’ ($\hat{\mu}$=-5.20, 95% CI [-6.56 to -3.85]; Δ = 6.65, 95% CI [4.83 to 8.46]), ‘limited imaginal capacity’ ($\hat{\mu}$=-0.89, 95% CI [-2.23 to 0.47]; Δ = 2.33, 95% CI [0.50 to 4.16]), ‘anxiety’ ($\hat{\mu}$=-4.77, 95% CI [-6.12 to -3.41]; Δ = 6.21, 95% CI [4.39 to 8.05]), ‘depression’ ($\hat{\mu}$=-5.87, 95% CI [-7.21 to -4.51]; Δ = 7.31, 95% CI [5.50 to 9.12]), and ‘health anxiety’ ($\hat{\mu}$=-7.31, 95% CI [-8.66 to -5.95]; Δ = 8.75, 95% CI [6.92 to 10.56]).

**Table S5**

*Estimates and Associated 95% Credibility Intervals of the Relevance Score for each TAS Item of the Difficulty Identifying Feelings Scale for each of the Constructs*

| Item | Difficulty identifying feelings  $\hat{\mu}$ [CI] | Difficulty  describing  feelings  $\hat{\mu}$ [CI] | Limited  imaginal  capacity  $\hat{\mu}$ [CI] | Externally-  oriented  thinking  $\hat{\mu}$ [CI] | Anxiety  $\hat{\mu}$ [CI] | Health anxiety  $\hat{\mu}$ [CI] | Depression  $\hat{\mu}$ [CI] |
| --- | --- | --- | --- | --- | --- | --- | --- |
| 1 | 7.08^*^  [5.68, 8.49] | 4.41^*^  [3.01, 5.82] | -2.99^*^  [-4.40, -1.58] | -4.89^*^  [-6.29, -3.49] | -3.26^*^  [-4.66, -1.87] | -5.99^*^  [-7.39, -4.59] | -4.41^*^  [-5.81, -3.00] |
| 2 | 3.09^*^  [1.69, 4.50] | 8.42^*^  [7.04, 9.70] | -1.39  [-2.80, 0.01] | -5.41^*^  [-6.82, -4.00] | -4.52^*^  [-5.93, -3.13] | -7.00^*^  [-8.41, -5.59] | -2.48^*^  [-3.88, -1.07] |
| 3 | -1.74^*^  [-3.24, -0.24] | -2.68^*^  [-4.19, -1.19] | -6.47^*^  [-7.98, -4.96] | -4.29^*^  [-5.79, -2.80] | 1.88^*^  [0.39, 3.38] | 6.54^*^  [5.02, 8.04] | -3.03^*^  [-4.53, -1.55] |
| 4 | 2.32^*^  [0.92, 3.75] | 4.29^*^  [2.89, 5.69] | -4.92^*^  [-6.33, -3.52] | -5.74^*^  [-7.15, -4.33] | -6.32^*^  [-7.72, -4.92] | -7.40^*^  [-8.81, -5.99] | -6.37^*^  [-7.78, -4.98] |
| 5 | -3.04^*^  [-4.27, -1.81] | -4.23^*^  [-5.46, -3.00] | -2.95^*^  [-4.17, -1.73] | -5.01^*^  [-6.22, -3.79] | -6.97^*^  [-8.21, -5.75] | -7.22^*^  [-8.45, -5.99] | -6.39^*^  [-7.62, -5.16] |
| 6 | 8.36^*^  [6.99, 9.66] | 4.38^*^  [2.99, 5.77] | -3.64^*^  [-5.03, -2.25] | -4.64^*^  [-6.04, -3.25] | -3.19^*^  [-4.57, -1.79] | -5.94^*^  [-7.33, -4.55] | -1.68^*^  [-3.06, -0.29] |
| 7 | 4.45^*^  [2.88, 6.01] | -0.13  [-1.68, 1.43] | -5.24^*^  [-6.80, -3.67] | -4.63^*^  [-6.19, -3.07] | -1.25  [-2.81, 0.32] | 2.59^*^  [1.03, 4.15] | -5.17^*^  [-6.74, -3.61] |
| 8 | -4.20^*^  [-5.62, -2.77] | -4.30^*^  [-5.72, -2.88] | -1.40  [-2.82, 0.01] | 1.48^*^  [0.06, 2.91] | -5.36^*^  [-6.76, -3.94] | -6.45^*^  [-7.86, -5.01] | -4.20^*^  [-5.62, -2.78] |
| 9 | 8.17^*^  [6.73, 9.57] | 3.99^*^  [2.54, 5.45] | -1.47^*^  [-2.91, -0.02] | -5.52^*^  [-6.97, -4.07] | -2.31^*^  [-3.77, -0.86] | -5.30^*^  [-6.75, -3.87] | -2.20^*^  [-3.66, -0.77] |
| 10 | -0.65  [-2.08, 0.77] | -1.81^*^  [-3.23, -0.39] | -4.88^*^  [-6.31, -3.46] | -3.86^*^  [-5.28, -2.44] | -5.34^*^  [-6.75, -3.92] | -6.87^*^  [-8.30, -5.44] | -4.47^*^  [-5.89, -3.05] |

*Note*: $\hat{\mu}$ = mu, estimated mean in the population, CI = 95% confidence interval, * *p* < 0.05, indicating a significant difference in score with the respective construct.

**Table S5 (continued)**

*Estimates and Associated 95% Credibility Intervals of the Relevance score for each TAS Item of the Difficulty Identifying Feelings Scale for each of the Constructs*

| Item | Difficulty identifying feelings  $\hat{\mu}$ [CI] | Difficulty  describing  feelings  $\hat{\mu}$ [CI] | Limited  imaginal  capacity  $\hat{\mu}$ [CI] | Externally-  oriented  thinking  $\hat{\mu}$ [CI] | Anxiety  $\hat{\mu}$ [CI] | Health anxiety  $\hat{\mu}$ [CI] | Depression  $\hat{\mu}$ [CI] |
| --- | --- | --- | --- | --- | --- | --- | --- |
| 11 | 3.29^*^  [1.94, 4.64] | 8.04^*^  [6.68, 9.37] | -1.97^*^  [-3.33, -0.60] | -4.49^*^  [-5.85, -3.13] | -4.98^*^  [-6.34, -3.62] | -7.43^*^  [-8.79, -6.06] | -4.43^*^  [-5.81, -3.06] |
| 12 | 1.58^*^  [0.09, 3.08] | 6.49^*^  [4.99, 7.99] | -2.15^*^  [-3.64, -0.65] | -3.38^*^  [-4.88, -1.90] | -4.90^*^  [-6.39, -3.38] | -5.99^*^  [-7.47, -4.50] | -3.46^*^  [-4.95, -1.97] |
| 13 | 7.60^*^  [6.04, 9.13] | 5.90^*^  [4.33, 7.46] | -2.30^*^  [-3.86, -0.74] | -3.62^*^  [-5.17, -2.07] | 1.04  [-0.51, 2.59] | -1.01  [-2.56, 0.55] | -0.33  [-1.88, 1.22] |
| 14 | 5.66^*^  [4.16, 7.16] | 3.32^*^  [1.83, 4.81] | -2.74^*^  [-4.22, -1.26] | -3.83^*^  [-5.31, -2.34] | -3.94^*^  [-5.43, -2.45] | -6.14^*^  [-7.62, -4.66] | -3.16^*^  [-4.64, -1.67] |
| 15 | 0.69  [-0.81, 2.19] | 2.38^*^  [0.88, 3.86] | -3.21^*^  [-4.71, -1.72] | 0.36  [-1.15, 1.86] | -4.43^*^  [-5.92, -2.93] | -7.17^*^  [-8.66, -5.68] | -4.70^*^  [-6.19, -3.21] |
| 16 | -4.87^*^  [-6.38, -3.36] | -5.02^*^  [-6.52, -3.50] | -2.67^*^  [-4.17, 1.15] | -1.15  [-2.66, 0.36] | -1.30  [-2.81, 0.21] | -4.41^*^  [-5.91, -2.89] | -3.21^*^  [-4.73, -1.70] |
| 17 | -0.01  [-1.53, 1.48] | 6.72^*^  [5.22, 8.24] | -6.39^*^  [-7.89, -4.88] | -5.85^*^  [-7.36, -4.34] | 0.42  [-1.08, 1.93] | -6.24^*^  [-7.76, -4.74] | -1.00  [-2.49, 0.51] |
| 18 | -3.75^*^  [-5.09, -2.43] | -4.49^*^  [-5.81, -3.15] | -4.80^*^  [-6.14, -3.47] | -5.41^*^  [-6.75, -4.08] | -4.02^*^  [-5.36, -2.70] | -6.95^*^  [-8.28, -5.62] | -4.76^*^  [-6.08, -3.43] |
| 19 | 0.67  [-0.59, 1.94] | -1.86^*^  [-3.13, -0.59] | -5.26^*^  [-6.52, -3.98] | -4.92^*^  [-6.19, -3.64] | -6.00^*^  [-7.27, -4.74] | -6.73^*^  [-8.00, -5.48] | -5.61^*^  [-6.88, -4.36] |
| 20 | -5.20^*^  [-6.56, -3.85] | -6.53^*^  [-7.89, -5.19] | -0.89  [-2.23, 0.47] | 1.45^*^  [0.09, 2.79] | -4.77^*^  [-6.12, -3.41] | -7.31^*^  [-8.66, -5.95] | -5.87^*^  [-7.21, -4.51] |

*Note*: $\hat{\mu}$ = mu, estimated mean in the population, CI = 95% confidence interval, * *p* < 0.05, indicating a significant difference in score with the respective construct.
